# Structural basis of binding of fluorescent, site-specific dansylated amino acids to human serum albumin

**DOI:** 10.1016/j.jsb.2010.10.004

**Published:** 2011-04

**Authors:** Ali J. Ryan, Jamie Ghuman, Patricia A. Zunszain, Chun-wa Chung, Stephen Curry

**Affiliations:** a Biophysics Section, Blackett Laboratory, Imperial College, Exhibition Road, London SW7 2AZ, United Kingdom; b Biomolecular Structure, Molecular Discovery Research, GlaxoSmithKline, Stevenage SG1 2NY, United Kingdom

**Keywords:** Human serum albumin, Dansylated amino acids, Protein-drug interactions, X-ray crystallography

## Abstract

Human serum albumin (HSA) has two primary binding sites for drug molecules. These sites selectively bind different dansylated amino acid compounds, which—due to their intrinsic fluorescence—have long been used as specific markers for the drug pockets on HSA. We present here the co-crystal structures of HSA in complex with six dansylated amino acids that are specific for either drug site 1 (dansyl-l-asparagine, dansyl-l-arginine, dansyl-l-glutamate) or drug site 2 (dansyl-l-norvaline, dansyl-l-phenylalanine, dansyl-l-sarcosine). Our results explain the structural basis of the site-specificity of different dansylated amino acids. They also show that fatty acid binding has only a modest effect on binding of dansylated amino acids to drug site 1 and identify the location of secondary binding sites.

## 1 Introduction

**Fig.1:**
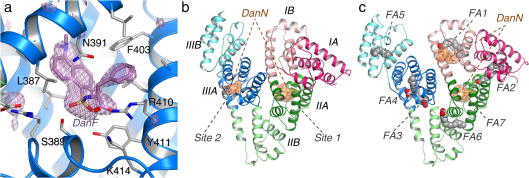
Location of binding sites for dansylated amino acids determined by X-ray crystallography. (a) Simulated annealing *F*_o_ − *F*_c_ omit map (contoured at 3*σ*) showing DanF bound to drug site 2 in sub-domain IIIA. DanF is shown in a stick representation with atoms coloured by atom-type: C – purple; O – red; and N – blue. Sub-domain IIIA is shown in a ribbon representation (blue). Selected protein side-chains are shown as sticks, with carbon atoms coloured grey. (b) Overview of DanN bound to drug sites 1 and 2 in HSA. The ligands are shown in a stick representation with a semi-transparent molecular surface. The protein secondary structure is shown coloured by sub-domain. This colour scheme is maintained throughout. (c) Overview of DanN bound to sub-domains IIA and IB in HSA–myristate. The seven fatty acid binding sites on the protein are labelled FA1–FA7. (For interpretation of the references to colour in this figure legend, the reader is referred to the web version of this article.)

With a typical circulating concentration of around 0.6 mM, HSA is the most abundant protein in blood plasma and serves as an important transporter molecule (Carter and Ho, 1994; Peters, 1995). The monomeric 67 kDa protein has three structurally homologous α-helical domains (I-III), each subdivided into sub-domains A and B (He and Carter, 1992) (Fig. 1). Despite these internal architectural similarities, HSA has several distinct binding compartments distributed asymmetrically across the protein and can accommodate a structurally diverse set of ligands. These ligands nevertheless share the general property of being poorly soluble hydrophobic compounds with anionic or electronegative features and include non-esterified fatty acids, thyroxine, uremic toxins, hemin and bilirubin (Peters, 1995). HSA greatly increases the solubilising capacity of plasma for its cargo compounds, allowing them to be present at near millimolar concentrations that are significantly in excess of their aqueous solubilities (Richieri and Kleinfeld, 1995; Spector, 1986). The binding capacity of HSA varies widely for different ligands. For example, there are up to seven binding sites for long-chain fatty acids, widely considered to be the primary ligand of the protein (Bhattacharya et al., 2000b; Curry et al., 1998); these sites, labelled FA1–FA7 (Fig. 1c), have variable binding affinities and are formed by residues from all six sub-domains (Kragh-Hansen et al., 2006; Simard et al., 2006, 2005) (Fig. 1). In contrast, there is only one primary site for large compounds such as hemin (Wardell et al., 2002; Zunszain et al., 2003) or bilirubin (Brodersen, 1980; Wells et al., 1982).

HSA can also bind drug compounds with similar physiochemical properties to its natural ligands and may significantly impact the kinetics of their distribution and elimination. Consequently, the investigation of HSA–drug interactions has long been of major interest to pharmaceutical scientists. One of the most important breakthroughs in this area happened in the mid-1970s when Sudlow and colleagues used a range of drugs and fluorescent dansylated amino acids in competition binding experiments to identify two primary drug-binding sites on HSA (Sudlow et al., 1975, 1976). Later crystallographic analysis of complexes of HSA with specific compounds confirmed that drug sites 1 and 2, as they came to be called, are located in sub-domains IIA and IIIA, respectively (He and Carter, 1992) (Fig. 1b). More recently, extensive crystallographic studies of the binding of drugs and drug-like molecules to HSA have confirmed the primacy of drug sites 1 and 2 but also identified additional binding sites across the protein (Bhattacharya et al., 2000a; Hein et al., 2010; Lejon et al., 2008; Petitpas et al., 2001, 2003; Zhu et al., 2008). Many of these additional sites are likely to be secondary binding pockets, which are probably occupied as a result of the high drug concentrations typically used to prepare HSA–drug complexes for crystallographic work, though sub-domain IB was recently identified as the primary binding site for the steroid antibiotic, fusidic acid (Zunszain et al., 2008).

These crystallographic investigations have also examined the structural impact of fatty acid binding on drug interactions with the protein, an important consideration for physiological drug binding which normally occurs in the presence of fatty acid. Long-chain fatty acids bind with high affinity to drug site 2 and will therefore competitively displace such compounds (Simard et al., 2006, 2005). In contrast, drug site 1 in sub-domain IIA has a low affinity for fatty acids and is unlikely to be occupied by this class of ligand under normal physiological conditions. However, the structure of the binding site is altered by binding of fatty acids to a high-affinity site nearby (FA2) Ghuman et al., 2005, an effect that is likely to account for some observed differences in drug binding in the presence of lipids (Birkett et al., 1977; Vorum and Honoré, 1996).

Although structural studies are a powerful way to locate ligand binding sites on HSA, they remain technically challenging and are hardly a convenient method for determining the binding location of novel drug compounds (Curry, 2009). However, the dansylated amino acids first used by Sudlow and colleagues to determine the number and specificity of the drug-binding sites on HSA remain useful tools for mapping drug-binding sites on the protein since they can be targeted to specific binding pockets and, being highly fluorescent, can be used in high-throughput competition binding assays (Sudlow et al., 1975, 1976). They have been in continuous use since their binding to HSA was first characterised over 30 years ago (Chen and Guo, 2009; Lissi et al., 2009; Mathias and Jung, 2007; Nakajou et al., 2003; Sakai et al., 2001; Thorarensen et al., 2007; Yamasaki et al., 1996).

The physicochemical properties of the amino acid side chains of the dansylated amino acids determine which drug-binding site they bind. Those with hydrophobic side chains (*e.g.* dansyl-l-phenylalanine (DanF) and dansyl*-*l-norvaline (DanNV)) are specific for drug site 2, as do dansylated amino acids such as dansylsarcosine (DanSRC) and dansyl*-*l-proline that have a methylated or blocked α-amino group (Sudlow et al., 1975, 1976). In contrast, dansylated amino acids with polar or charged side chains bind preferentially to drug site 1 (*e.g.* dansyl*-*l-asparagine (DanN), dansyl*-*l-arginine (DanR) and dansyl*-*l-glutamate (DanE)) (Sudlow et al., 1975, 1976).

Despite their evident utility in drug binding experiments, there have been no investigations of the structural aspects of the interactions of dansylated amino acids with HSA. Here we report the crystal structures of HSA in complex with six different dansylated amino acids that are specific either for drug site 1 (DanN, DanE, DanR) or drug site 2 (DanF, DanNV and DanSRC). Our results explain the structural basis of the site-specificity of these compounds. They also reveal secondary binding sites for some compounds (DanN, DanF). In the case of DanN, we also show that, although fatty acid binding induces restructuring of one flanking wall of drug site 1, it has minimal impact on the binding of the dansylated compound to the pocket.

These structures represent the first case of a group of structurally related drug-like compounds being co-crystallised with HSA. They provide new insights into the specificity determinants of the drug-binding pockets and should help future investigations of HSA–drug interactions.

## 2 Materials and methods

Purified recombinant HSA was kindly provided by Prof. Eishun Tsuchida (Waseda University) and, as Recombumin™, by Delta Biotechnology Ltd. (Nottingham, England). HSA was first defatted (Sogami and Foster, 1968) and run on a Superdex S75 HR10/30 gel filtration column to remove contaminating HSA dimers to ensure a purely monomeric preparation of the protein, as described previously (Bhattacharya et al., 2000a; Curry, 2009). HSA used in the crystallisation of HSA–myristate complexes did not require defatting (Curry et al., 1998).

All dansylated amino acids were obtained from Sigma–Aldrich at the highest available purity. For preparation of complexes with defatted HSA the dansylated amino acids were typically dissolved at 100 mM in dimethylsulphoxide (DMSO) (25 mM in the case of DanN). Complexes with HSA were prepared by incubating the drug at a 5:1 mol ratio with HSA, typically by mixing 500 μL 1.5 mM HSA in 20 mM potassium phosphate, pH 7.0 with 37.6 μL 100 mM dansylated amino acid and incubating at room temperature for at least 1 h. The DMSO concentration was then reduced to <0.1% (v/v) by repeated cycles of dilution in phosphate buffer and re-concentration, as described previously (Ghuman et al., 2005).

The crystallisation conditions used for both HSA–dansylated amino acid and HSA–myristate complexes were the same as those that have been published (Bhattacharya et al., 2000a,b; Ghuman et al., 2005). To prepare ternary HSA–myristate–dansylated amino acid complexes for DanN, DanE and DanR HSA–myristate crystals were harvested into artificial mother liquor (typically 36% PEG 3350, 50 mM potassium phosphate pH 7.0) and then incubated for 24–48 h in the same solution containing 5 mM of the dansylated amino acid, following a protocol developed earlier for HSA–myristate–drug ternary complexes (Ghuman et al., 2005).

All crystals were mounted in thin walled glass capillaries for data collection at room temperature. Data were collected at beamline 9.6 at the SRS Daresbury (UK) and at beamlines X13 and X11 at EMBL/DESY Hamburg (Germany). The data were processed and scaled using the CCP4 software suite (Collaborative Computer Project, 1994). In all cases the HSA–dansylated amino acid complexes co-crystallised isomorphously in the P1 unit cell observed previously for defatted HSA crystals (Bhattacharya et al., 2000a). Diffraction data were phased by molecular replacement using the structure of defatted HSA complexed with 3-carboxy-4-methyl-5-propyl-2-furan-propanoic acid (PDB: 2BXA Ghuman et al., 2005), stripped of its ligands. Crystals of HSA–myristate–dansylated amino acid complexes had the C2 unit cell expected for HSA–myristate crystals (Bhattacharya et al., 2000b). In this case the diffraction data were phased using the structure of the HSA–myristate complex (PDB: 1E7G Bhattacharya et al., 2000b), again minus its ligands. Model building was performed using O (Jones et al., 1991). Coordinates for the dansylated compounds were generated by modifying the structure of dansylglycine (Antolini et al., 1986), which was obtained from the small molecule Cambridge structural database via the Chemical Database Service (Fletcher et al., 1996). All models were refined using CNS (Crystallography and NMR System) version 1.2 (Brünger et al., 1998).

## 3 Results

### 3.1 Structure determination

The dansylated amino acids DanN, DanSRC, DanNV and DanF were co-crystallised with defatted HSA at a mole ratio of 5:1 (compound:protein) and yielded crystals in space-group P1 with unit cell dimensions that are essentially identical to crystals of HSA–drug complexes obtained previously (Ghuman et al., 2005); these have two HSA molecules in the asymmetric unit, giving two independent structures of the HSA–dansylated amino acid complex. HSA–myristate complexes were crystallised in space-group C2 (with one molecule per asymmetric unit) as described previously (Bhattacharya et al., 2000b); DanN, DanE and DanR were added by soaking these crystals in solutions containing 5 mM of the fluorescent compound (see Section 2). High concentrations of dansylated amino acids were used in soaking and co-crystallisation experiments to ensure full occupancy of the primary binding sites.

In all seven cases Fourier difference electron density maps calculated following phasing by molecular replacement (Section 2) clearly indicated the location of at least one bound dansylated amino acid molecule. In combination with inspection of the chemistry of the binding environment, this provided an unambiguous determination of the drug binding conformation (Fig. 1a). For several of the complexes, density indicating the occupancy of secondary binding sites was also observed; where this occurred, an additional molecule of the dansylated amino acid was built into the model (Fig. 1b and c). Refinement of the different complexes against data with high-resolution limits in the range 2.4–2.9 Å produced models with good stereochemistry and *R*_free_ values in the range of 24.8–25.6%. Full data collection and refinement statistics are given in Table 1.

### 3.2 Drug site 1 specific dansylated amino acids

**Fig.2:**
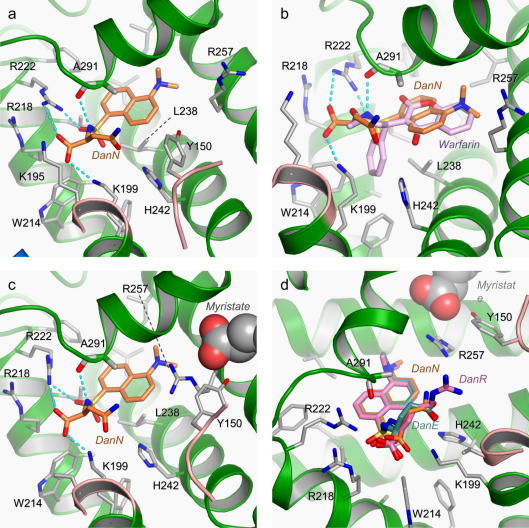
Binding interactions made by dansylated amino acids (DanN, DanE, DanR) in drug site 1 (sub-domain IIA) of HSA in the absence and presence of fatty acid. (a) Binding of DanN (coloured by atom type as in Fig. 1b). Hydrogen bonds are shown as dotted cyan lines. (b) Comparison of the binding of DanN and warfarin (Petitpas et al., 2001). The view is rotated slightly to the left compared to panel a; the polypeptide containing Y150 has been removed for clarity. (c) Binding of DanN to HSA in the presence of myristate (shown as CPK spheres, coloured by atom type: carbon – grey and oxygen – red). (d) Comparison of the binding of DanN, DanE and DanR to HSA–myristate. (For interpretation of the references to colour in this figure legend, the reader is referred to the web version of this article.)

The structure of DanN bound to defatted HSA (Fig. 2a) shows that the dansyl group binds in the centre of drug site 1 in sub-domain IIA between the side-chains of Ala-291 and Leu-238; the plane of the dansyl ring is approximately perpendicular to the line connecting the *C*_α_ atoms of this pair of residues. This group therefore binds in the same position and planar orientation as observed for the major aromatic moieties of many other site 1 drugs, including warfarin and azapropazone (see Section 4) (Fig. 1b) Ghuman et al., 2005; Petitpas et al., 2001. The dimethylamine group of the dansyl ring occupies what was previously termed the right hand sub-chamber (Ghuman et al., 2005), close to the side-chains of Leu-260, Ala-261, Ser-289 and the aliphatic portion of Arg-257. The other end of the molecule is positioned close to the polar entrance to the binding pocket. The amide nitrogen of the sulphonamide moiety forms a hydrogen bond with the backbone carbonyl of Ala-291, while one of the two SO_2_ oxygen atoms receives a hydrogen bond from the side-chain of Arg-222 on one side of the pocket entrance. The carboxylate group of the amino acid makes two salt bridges with basic side-chains, Lys-199 and Arg-222. In addition the carbonyl group of the Asn side-chain of DanN is hydrogen bonded to Lys-199. Unusually among drugs that have been observed through crystallography to bind to site 1, DanN does not make a hydrogen bond with the side-chain hydroxyl of Tyr-150 in defatted HSA (Ghuman et al., 2005).

Surprisingly, since DanN is classified as a compound that is specific for drug site 1, the electron density map for the HSA–DanN complex clearly shows the presence of a second DanN molecule, bound to drug site 2 in sub-domain IIIA. This is likely to be a lower affinity binding site for DanN since competition binding experiments performed using a 1:10 molar ratio of DanN to HSA only detected significant displacement by drugs specific for drug site 1 in sub-domain IIA (Sudlow et al., 1976). This secondary site for DanN is probably only occupied because of the high concentrations of the ligand used in the preparation of the complex (see Section 2). The details of binding of DanN to drug site 2 will be described below in conjunction with the comparison of the binding interactions made by the three dansylated amino acids that are specific for that site (DanF, DanNV, DanSRC).

In the structure of the ternary HSA–myristate–DanN complex there are six molecules of fatty acid, bound to fatty acid sites 1–6 (FA1–6) Bhattacharya et al., 2000b; Curry et al., 1999 (Fig. 1c). A molecule of DanN is observed bound to drug site 1 but the structure reveals that binding of fatty acid to sites FA3 and FA4 in sub-domain IIIA, which overlap extensively with drug site 2, prevented DanN from binding to this region of the protein. In contrast, although drug site 1 overlaps with site FA7, it has low affinity for fatty acid (Simard et al., 2006) and under our experimental conditions the dansylated amino acid is able to displace the lipid. These observations are consistent with drug site 1 being a high-affinity site for DanN (Fig. 1b and c).

Interestingly, a second molecule of DanN is observed bound in the HSA–myristate–DanN complex in direct contact with the myristate molecule in site FA1 (sub-domain IB) (Fig. 1c; Supp. Fig. 1). Since DanN is not observed to bind to this site in the absence of fatty acid, this binding appears to be the result of a cooperative interaction between the dansylated compound and the fatty acid.

Although the binding of myristate to HSA causes significant conformational changes in the protein that have a large impact on drug site 1 (Ghuman et al., 2005), the structure of the HSA–myristate–DanN complex reveals that fatty acid binding results in only very modest changes in the mode of binding of the dansylated amino acid to this site (Fig. 2c). The conformational changes are located on one flank of the binding pocket within sub-domain IIA and principally involve re-orientation or relocation of the side-chains of Tyr-150, Glu-153, Gln-196 and Arg-257. These form a fairly polar flanking wall but, apart from Arg-257, do not contact DanN in the binary HSA–DanN complex. As a result the changes in the position of the bound DanN upon addition of myristate are minimal. The compound is shifted slightly (0.4 Å) towards the polar mouth of the pocket but maintains the hydrogen bonds that were observed in the defatted HSA–DanN complex (although the hydrogen bond between the side-chain of Arg-222 and the sulphonamide oxygen is stretched from 2.6 Å to 3.3 Å). There is a slight adjustment of the position of the Asn side-chain in DanN but the hydrogen bond with Lys-199 is preserved.

The structures of the HSA–myristate–DanE and HSA–myristate–DanR ternary complexes reveal exactly the same pattern of ligand binding as was observed for DanN: sites FA1–6 are occupied by lipid and molecules of the dansylated amino acid are found in sub-domain IIA (drug site 1) and in contact with myristate in sub-domain IB. The dansyl groups of DanE and DanR bind in the same positions as observed for DanN in both of these sites and make the same specific hydrogen bonding interactions with the protein. It seems clear that this moiety provides important anchoring interactions with the protein.

As might be expected, the interactions made by the amino acid side-chains of the dansylated compounds show more variability. Although the Glu side-chain of DanE interacts with Lys-199 (as observed for Asn side-chain of DanN), the Arg side-chain of DanR adopts a more extended conformation, allowing the guanidinium group to make a salt-bridge—albeit a long one (3.5 Å)—with the side-chain of Glu-153 (Fig. 2d).

What would be the interactions made by DanE and DanR in drug site 1 in the *absence* of fatty acid? Since the mode of binding of DanN was found to be essentially unaffected by fatty acid binding to HSA and DanE makes essentially the same set of interactions with the drug binding pocket, it seems likely that bound conformation of DanE would be unchanged in defatted HSA. It is more difficult to predict the binding mode for DanR, since it is observed to interact with the side-chain of Glu-153, a residue that is normally tied up in an interaction with His-288 in defatted HSA (Ghuman et al., 2005). However, modelling of the bound conformation of DanR into drug site 1 in the defatted protein shows that there is ample room for the Arg side-chain and the possibility of a specific hydrogen bond interaction with the side-chain of Ser-192. It is therefore likely to be able to bind to drug site 1 in the absence of fatty acid.

The ability of drug site 1 in HSA to bind dansylated amino acids with a range of polar side-chains appears to be due to the large size of the pocket, which can accommodate medium and large-sized side-chains *and* to the fact that it possesses polar groups that provide counter-charges to stabilise the side-chains on the dansylated compounds (Fig. 2d).

Comparison of the binding of DanN, DanE and DanR to sub-domain IB in the HSA complexes with myristate reveals that the dansyl moieties bind in exactly the same way—in contact with the myristate molecule (site FA1)—but that the amino acid groups are all exposed to solvent and their side-chains make no significant contact with the protein (Supp. Fig. 1). This secondary binding site is also observed to be occupied in HSA–myristate by other compounds that are specific for drug site 1 (*e.g.* warfarin, indomethacin and salicylate) (Ghuman et al., 2005; Petitpas et al., 2001; Zhu et al., 2008). It is probably not physiologically significant for these compounds or dansylated amino acids because their binding is dependent on contacts with bound lipid which is unlikely to be present under normal conditions due to the low affinity of site FA1 for fatty acids (Simard et al., 2006).

### 3.3 Drug site 2 specific dansylated amino acids

Since fatty acid binding sites FA3 and FA4 in sub-domain IIIA overlap extensively with drug site 2, the binding of dansylated compounds specific for drug site 2 was examined by co-crystallising them with defatted HSA (Section 2). Three binary complexes were investigated; two of these had large hydrophobic side-chains on the amino acid group (DanF, DanNV), while the third (DanSRC)—a derivative of dansylglycine—has a methylated amide group.

**Fig.3:**
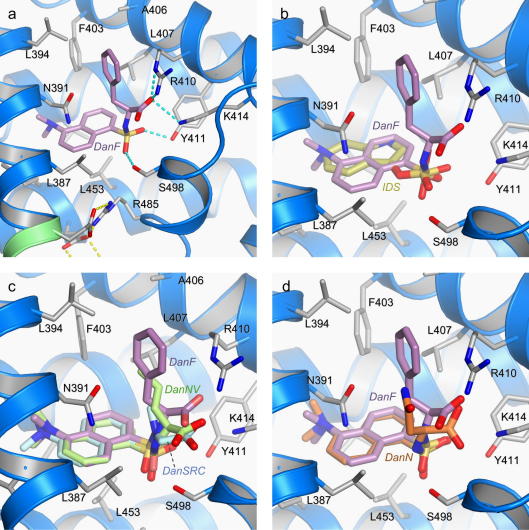
Binding interactions made by dansylated amino acids (DanSRC, DanNV, DanF and DanN) in drug site 2 (sub-domain IIIA) of HSA. (a) Binding of DanF (coloured by atom type as in Fig. 1a). Hydrogen bonds are shown as dotted cyan lines. (b) Comparison of the binding of DanF and indoxyl sulphate (IDS) Ghuman et al., 2005; carbon atoms in IDS are coloured dark yellow. (c) Comparison of the binding of DanF, DanNV and DanSRC to drug site 2 of HSA. (d) Comparison of the binding of DanF and DanN to drug site 2 of HSA. (For interpretation of the references to colour in this figure legend, the reader is referred to the web version of this article.)

As was observed for dansylated amino acids bound to drug site 1, the dansyl group provides the most important anchoring interactions for binding DanF, DanNV and DanSRC in drug site 2 (Fig. 3). In each case the dansyl group occupies the same position in the binding site pinned between the side-chains of Asn-391 and Phe-403 on one side and Leu-453 on the other. The dimethylamine group occupies the deepest part of the pocket, contacting the apolar side-chains of Ile-388, Val-433 and the disulphide bond formed by Cys-393 and Cys-438. The dansyl group occupies the same part of the pocket as the major aromatic moieties of diazepam, diflunisal, ibuprofen, propofol and indoxyl sulphate (Bhattacharya et al., 2000a; Ghuman et al., 2005). Because of the presence of the dimethylamine group the fused ring system of the dansyl group is displaced relative to the bound position of the indole ring of indoxyl sulphate (Fig. 3b); nevertheless the SO_2_ group of the dansylated amino acids occupies the same position as the SO_3_ group of indoxyl sulphate and interacts with some of the same polar groups near the mouth of the pocket. For all three dansylated amino acids the oxygen atom on one side of the SO_2_ group makes a hydrogen bond with the side-chain of Tyr-411. The other oxygen atom of the SO_2_ makes a 3.0 Å hydrogen bond to the hydroxyl group of Ser-489 in the case of DanF; however, the distance between these groups is stretched to 3.7 Å for DanNV and to 4.1 Å for DanSRC. The variability in the orientations of the SO_2_ groups arises because of the differential positioning of the amino acid groups of the different dansyl compounds (Fig. 3c); this also has some impact on the position of their amino acid carboxylate groups, though they all maintain a single salt-bridge to the side-chain of Lys-411.

In all three structures accommodation of the amino acid moiety results in displacement of the side-chain of Arg-410 from the position that it is observed to occupy in defatted HSA or in complexes with smaller site 2 drugs such as diflunisal, ibuprofen or indoxyl sulphate, which rely on the Arg side-chain for at least one hydrogen bond interaction (Bhattacharya et al., 2000a; Ghuman et al., 2005). In the presence of DanF, DanNV or DanSRC, the side-chain of Arg-410 is rotated towards the solvent or becomes disordered. This rotation reveals a hydrophobic flanking wall of drug site 2 that has not previously been noted but which contacts the apolar side-chains of DanF and DanNV and the methyl group added to the sulphonamide in DanSRC. The main contacts made by these apolar functional groups are with the hydrophobic side chains of Leu-396, Phe-403, Ala-406, Leu-407 and the apolar stem of Arg-410 (Fig. 3b,c). However, this hydrophobic flank of drug site 2 is not sufficient for complete burial of the side-chains of the dansylated amino acids, which remain partly exposed to solvent. This partial solvent exposure of the side chains binding within this pocket may explain why the affinity of DanF for this site is similar to that of DanSRC even though DanSRC has no side chain (Sudlow et al., 1975).

As mentioned above, drug site 2 is a secondary site for the binding of the site 1 specific compounds, DanN. The dansylated moiety of DanN binds to the pocket in exactly the same way as observed for DanF, DanNV and DanSRC (Fig. 3d). However, the polar Asn side-chain of DanN is rotated relative to the conformations observed for the side-chains of DanF and DanNV and does not make contact with the hydrophobic flank formed by Leu-396, Phe-403, Ala-406, Leu-407. Although the Asn side-chain can make a single, elongated hydrogen bond (3.2 Å) with the side-chain of Asn-391 (Fig. 3d) this is appears insufficient to overcome its inability to make favourable apolar contacts with the pocket and probably accounts for the lower affinity of DanN for drug site 2 (Sudlow et al., 1975).

Unexpectedly we also observed a second molecule of DanF in the HSA–DanF complex, bound to drug site 1 (Supp. Fig. 2). In this case, however, it adopted a binding conformation that was very different from that observed for dansylated compounds that are specific for drug site 1. Instead of pinning the dansyl moiety in position — in the manner observed for DanN, DanE and DanR — the side-chains of Leu-238 and Ala-291 are found to hold the Phe side-chain of DanF. The dansyl group of DanF is re-located to the lower part of drug site 1, which was previously observed to be occupied by indomethacin (Ghuman et al., 2005); as with indomethacin, the binding of DanF induces a rotation of the side-chain of Trp-214 (∼140° about *x*_2_). Since DanF was found not to be displaced by binding of drugs specific to site 1 (Sudlow et al., 1976), we conclude that the affinity of this compound for site 1 is much less than its affinity for drug site 2.

It should be noted that the electron density maps also suggest that a molecule of DanNV may also bind weakly to drug site 1. However, although there is broken density to indicate low occupancy binding of DanNV to the pocket, it was too weak to allow reliable model building and the molecule was omitted from the refined structure.

## 4 Discussion and conclusions

Thirty-five years after the establishment of dansylated amino acids as useful fluorescent markers for the two primary drug-binding sites on HSA (Sudlow et al., 1975), we have used X-ray crystallography to examine the structural basis for their site selectivity. The results presented here provide a clear explanation for the specificity of the dansylated amino acids characterised in the original work of Sudlow and colleagues (Birkett et al., 1977; Sudlow et al., 1975, 1976).

Dansylated amino acids specific for drug site 1 in HSA generally have polar side-chains which can be uncharged or positively or negatively charged, although dansyl-l-α-alanine or dansylglycine—which have small apolar side-chains—are also specific for this site (Fig. 2). Our results reveal that such compounds bind preferentially to drug site 1 since this pocket can accommodate the dansyl moiety in its central hydrophobic compartment in an orientation that allows the pocket to make specific hydrogen-bond interactions to the SO_2_ and amide groups of the sulphonamide moiety and to the common carboxylate group of the amino acid. Moreover, these anchoring interactions allow the polar amino acids groups of the ligand to be positioned within a hydrophilic sub-chamber that is both large enough to accommodate the variably-sized side-chains and contains counter-charges that favour their binding. Although the polar flanking wall of drug site 1 which interacts with the amino-acid side chains alters in composition upon fatty acid binding, this was found to have a minimal effect on the interactions made by the Asn side-chain of DanN; no steric clashes are introduced and the physicochemistry of the wall remains predominantly polar.

The bound orientation that is common to DanN, DanE and DanR is unlikely to be adopted by dansylated amino acids that have large apolar side-chains since this would place the side-chain in an unfavourable binding environment. Accordingly, we did not observe electron density to indicate binding of DanF or DanNV in this orientation in drug site 1.

The presence of a conserved hydrogen bond formed from the central amide group of the dansylated amino acids to the main-chain carbonyl oxygen of Ala-291 in site 1 explains why DanSRC does not bind to this pocket on HSA (Fig. 2a). Since the amide group in DanSRC is blocked by a methyl group, it cannot make this hydrogen bond interaction; indeed, our structural results show that methylation of the amide would create a steric clash with Ala-291 preventing the dansyl group of DanSRC from adopting the conformation favoured by site 1 specific compounds such as DanN, DanE and DanR (Sudlow et al., 1975, 1976). Similarly, blocking of the amide by a hydrophobic group in dansyl-l-proline, which is structurally similar in this respect to DanSRC, probably accounts for its inability to bind to site 1 and its preference for site 2 (Sudlow et al., 1975, 1976).

The central chamber in drug site 1 that is occupied by the dansyl groups of DanN, DanE and DanR overlaps extensively with the binding loci of many different drugs that are specific for that site, including azapropazone, phenylbutazone, oxyphebutazone, warfarin and iodipamide (Ghuman et al., 2005; Petitpas et al., 2001) (Fig. 2b). This readily explains the displacement of the fluorescent dansylated amino acids in drug competition binding assays (Sudlow et al., 1975, 1976). However, our results are not consistent with an earlier report, based on competition binding experiments, which concluded that the portion of drug site 1 bound by DanN did not overlap with the binding sites for warfarin or iodipamide (Yamasaki et al., 1996). The reason for the discrepancy between the structural and binding data is not entirely clear at present; one possibility is that the interpretation of the binding data may have taken full account of the presence of secondary binding sites for these compounds.

Even drugs that do not bind primarily to the central chamber may be expected to displace dansylated compounds from drug site 1. Indomethacin binds in the lower compartment of site 1 which is generally not occupied by polar dansylated amino acids (Ghuman et al., 2005); nevertheless superposition of the structures suggests that the carboxylate group of the drug would make a steric clash with the carboxylate moiety of the dansylated compounds. This drug would therefore be predicted to displace the reporter compounds.

Fatty acid binding is reported to cause up to a fivefold enhancement of the fluorescence of dansylated compounds that bind to drug site 1 (Birkett et al., 1977; Sudlow et al., 1976). Since the bound orientation of DanN was not observed to be greatly altered in the presence of fatty acids, this fluorescence enhancement appears to arise because of changes in the immediate environment of the dansyl group, due to the restructuring of polar flank of the binding site. A major factor may be the re-positioning of the guanidium group of Arg-257, which moves from 8 Å to 4 Å away from the dansyl group of DanN upon fatty acid binding (Fig. 2a and c).

In drug site 2 the dansylated moiety is again observed to be responsible for establishing a common mode of binding by securing the compound within the main hydrophobic compartment of the drug pocket (Fig. 3). In this case the hydrophobic side-chains of the amino acids are thereby placed in contact with an apolar flanking wall that is revealed by the displacement of Arg-410 upon binding of DanF, DanNV or DanSRC.

As found for drug site 1 the location of the dansyl group of the dansylated amino acid bound to drug site 2 overlaps extensively with the positions occupied by site 2 drugs or toxins such as ibuprofen, diflunisal, diazepam, indoxyl sulphate or propofol, readily accounting for the utility of DanF, DanNV and DanSRC as specific markers for drug site 2 (Sudlow et al., 1975, 1976) (Fig. 3b). It is interesting to note that other apolar dansylated amino acids, particularly dansyl-l-valine and dansyl-l-leucine that have branched side-chains, bind more weakly to drug site 2 *and* exhibit some binding to drug site 1 (Sudlow et al., 1976). At present it remains a puzzle why these compounds are significantly less specific for drug site 2.

Although the polar DanN was also observed to bind to drug site 2 under our experimental conditions, it cannot exploit the apolar feature of the pocket (Fig. 3d). This suggests it binds weakly, consistent with the observation that binding of the fluorescent compound was unaffected by site 2 specific drugs such as flubiprofen or ibuprofen under experimental conditions that were sufficient to detect displacement by site 1 specific compounds (*e.g.* warfarin) Sudlow et al., 1976. It is reasonable to assume that DanN can continue to be used as a specific marker for drug site 1.

## Figures and Tables

**Table 1:** Data collection and refinement statistics.

	Dansyl-sarcosine	Dansyl-Norvaline	Dansyl-Phe	Dansyl-Asn	Dansyl-Asn	Dansyl-Arg	Dansyl-Glu
Myristate	−	−	−	−	+	+	+
*Data collection*							
Space group	P1	P1	P1	P1	C2	C2	C2
Resolution range (Å)	52.0–2.9	49.6–2.45	44.5–2.4	35.1–2.6	22.5–2.4	32.6–2.65	21.4–2.7
*N*_refs_	27,597	42,048	49,448	37,571	25,305	19,137	18,820
Multiplicity^a^	1.9 (1.9)	1.8 (1.9)	1.8 (1.8)	2.0 (2.0)	1.9 (1.9)	2.7 (2.7)	2.8 (2.8)
Completeness (%)^a^	96.9 (98.4)	90.6 (85.6)	88.0 (78.2)	97.7 (97.4)	96.3 (96.7)	97.4 (99.))	98.5 (99.3)
*R*_merge_ (%)^a^^,^^b^	11.2 (37.1)	7.2 (51.0)	8.4 (38.2)	4.6 (38.5)	4.0 (31.4)	6.3 (40.0)	5.9 (32.3)
*I*/*σ*_I_	6.6 (2.1)	9.7 (2.9)	8.3 (1.4)	10.4 (2.3)	11.4 (2.6)	10.6 (2.8)	12.1 (3.6)

*Refinement*							
No. atoms (exc. H)	8285	8481	8440	8327	4775	4607	4630
No. of waters	26	28	18	35	39	23	32
*R*_model_ (%)^c^	22.0	22.8	22.8	20.7	20.6	19.1	19.0
*R*_free_ (%)^d^	25.6	25.7	25.6	24.8	25.0	25.2	25.1
RMS_bonds_ (Å)	0.009	0.009	0.009	0.007	0.007	0.007	0.007
RMS_angles_ (°)	1.29	1.23	1.26	1.22	1.23	1.22	1.24
Average B-factor (Å^b^)	73.0	75.3	73.4	64.8	54.4	56.2	54.0
Ramachandran plot^e^ (%core/allowed)	87.3/12.2	88.6/10.8	89.2/10.1	89.7/9.7	91.9/7.9	91.7/7.9	90.1/9.9
PDB ID	2*xvq*	2*xw*1	2*xw*0	2*xvu*	2*xvv*	2*xvw*	2*xsi*

a Values for the outermost resolution shell are given in parentheses.

b *R*_merge_ (%) = 100 × ∑_h_ ∑_j_ |*I*_hj_ − *I*_h_|/*∑*_h_*∑*_j_*I*_hj_, where *I*_h_ is the weighted mean intensity of the symmetry related reflections *I*_hj_.

c *R*_model_ (%) = 100 × *∑*_hkl_ |*F*_obs_ − *F*_calc_|/*∑*_hkl_*F*_obs_, where *F*_obs_ and *F*_calc_ are the observed and calculated structure factors, respectively.

d *R*_free_ (%) is the *R*_model_ (%) calculated using a randomly selected 5% sample of reflection data omitted from refinement.

e Numbers determined using Procheck (Laskowski et al., 1993).
