# Supplementary material for: Structural basis of binding of fluorescent, site-specific dansylated amino acids to human serum albumin
Source: J Struct Biol. 2011 Apr;174(1):84–91. doi: 10.1016/j.jsb.2010.10.004 (PMC3073228; doi:10.1016/j.jsb.2010.10.004)
Supplement: Supplementary data 1 [file mmc1.doc]

### SUPPLEMENTARY INFORMATION FOR:

### Structural basis of binding of fluorescent, site-specific dansylated amino acids to human serum albumin

Ali J. Ryan^1,#^, Jamie Ghuman^1^, Patricia A. Zunszain^1,†^, Chun-wa Chung^2^ and Stephen Curry^1*^

Supplementary Figure 1: Binding of DanN, DanE and DanR to a secondary site in sub-domain IB of HSA in the presence of myristate.

Supplementary Figure 2: Binding of DanF to a secondary site in sub-domain IIA (drug site 1) of HSA.


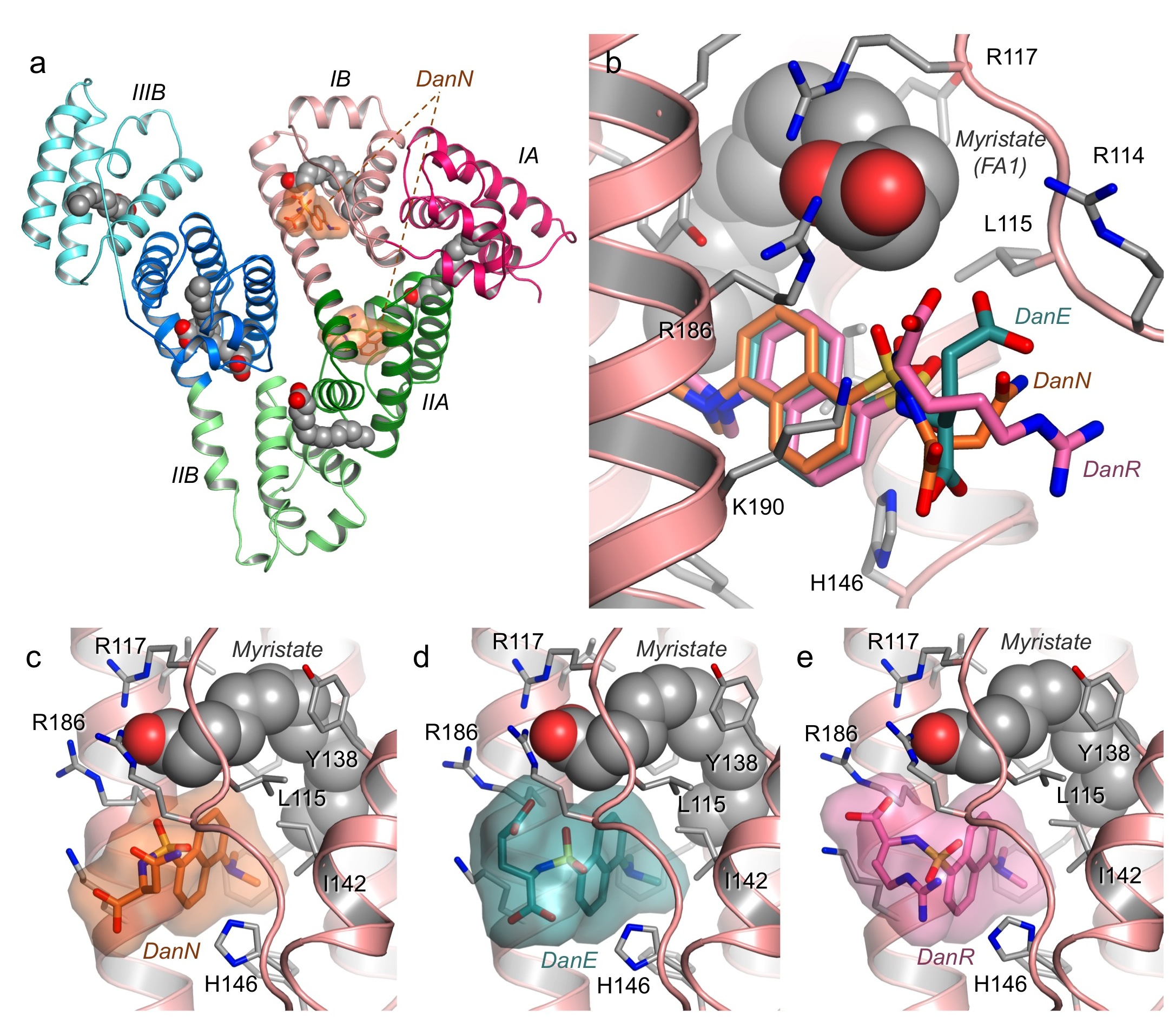


**Supplementary Figure 1:** Binding of DanN, DanE and DanR to a secondary site in sub-domain IB of HSA in the presence of myristate. (a) Overview of binding of DanN to its primary (IIA) and secondary (IB) sites on HSA-myristate. (b) Detailed comparative view of DanN, DanE and DanR binding to sub-domain IB. Dansylated amino acids are shown as sticks coloured by carbon atom (Dan N - orange; DanE - teal; DanR - pink); myristate is shown as CPK spheres. In the case of DanR, electron density for the amino-acid side chain is particularly weak so its position is not well determined. Close-up views of (c) DanN, (d) DanE and (e) DanR binding adjacent to myristate in sub-domain IB.


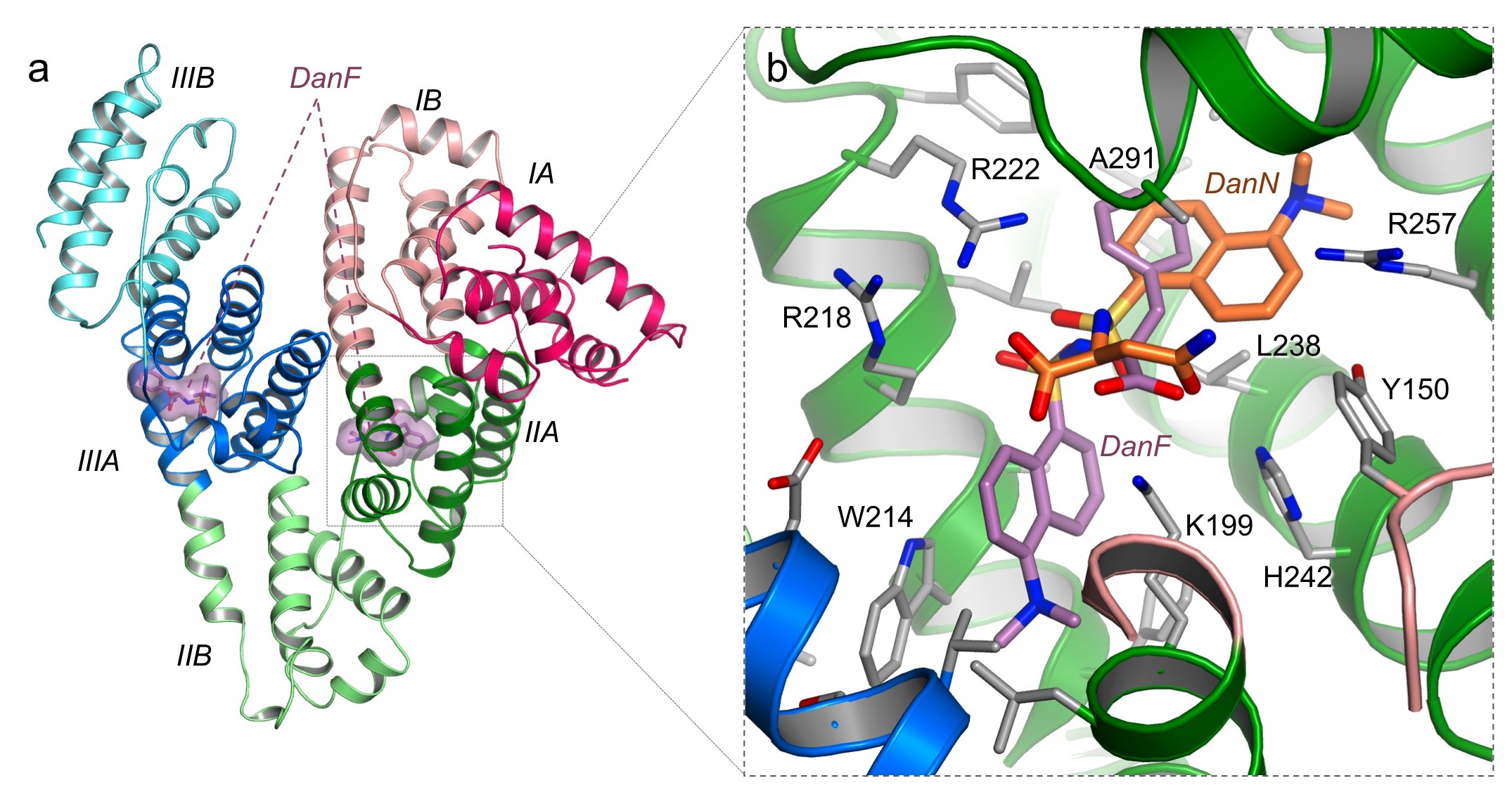


**Supplementary Figure 2:** Binding of DanF to a secondary site in sub-domain IIA (drug site 1) of HSA. (a) Overview of binding of DanF to its primary (IIIA) and secondary (IIA) sites on HSA-myristate. (b) Detailed comparative view of DanF and DanN binding to sub-domain IIA. Dansylated amino acids are shown as sticks coloured by carbon atom (DanF - purple; DanN - orange).
